# Stickiness of the blues: Chronicity of depression

**DOI:** 10.4103/0019-5545.49445

**Published:** 2009

**Authors:** G. Swaminath

**Affiliations:** Department of Psychiatry, Dr. B. R. Ambedkar Medical College, Kadugondanahalli, Bangalore - 560 045, Karnataka, India

“You cannot prevent the birds of sadness from passing over your head, but you can prevent their making a nest in your hair”

Chinese Proverb

## SONG SUNG BLUE

Depression is an important global public-health issue. This is because of the relatively high lifetime prevalence ranging from 2 to 15% and also because it is associated with substantial disability.[1] After the development of the metric Disability Adjusted Life Years (DALYs) as a measure of non-fatal outcomes of many diseases, depression came to be considered a disease with significant burden. The basic concept was that the years lost due to the disease would be added to the Years Lived with the Disability (YLD) due to the disease itself.[2] Rated the fourth leading cause of disease burden in 2000, depression accounted for 4.4% of total disability adjusted life years (DALYs).[3] It is also responsible for the greatest proportion of disease burden attributable to non-fatal health outcomes, accounting for almost 12% of total years lived with disability worldwide.[1] Without treatment, depression has the tendency to assume a chronic course, be recurrent, and over time to be associated with increasing disability.[4] Early detection, proper management with proactive follow-up could reduce the socio economic burden of depression, and help rationalise health care rather than ration it.[5]

In comparison with other chronic diseases such as diabetes, asthma, angina and arthritis in terms of decrement in health status, depression impairs health status to a substantially greater degree [3]. A significant number of patients with chronic diseases have comorbid depression. The presence of depression and its treatment is clearly related to the outcome of these chronic diseases. In addition, comorbidity with depression significantly worsens the health state of people with chronic diseases. Interestingly, comorbid depression worsened health status in chronic diseases when compared with the health status in the chronic diseases either singly or in combination, but without depression.[3] This puts depression on par, if not higher than other chronic diseases in damaging health, so health care providers should carefully note the presence of depression in view of the marked health effects.[3] This caution has been reiterated in an National Institute of Mental Health (NIMH) research paper on comorbidity: “Finding ways to improve detection and treatment of depression in the primary-care setting is an important NIMH research priority”.[6]

## A MEDICINE FOR MELANCHOLY

Though depression responds excellently to pharmacologic and behavioral treatments, both individually and in combination, it remains, stigmatized, under-recognized, and under-treated in primary care settings.[4] Depression lasts for a median of eight weeks (mean 16 weeks), with only 5% not responding within a year[7] owing to the increased frequency of spontaneous remission, the sensitivity of depressed patients to treatment as well as the encouraging effects of being in treatment.[4] After improvement 50% relapse within one year and most within two years. Of those patients who have one episode of major depression, 50-85% will go on to have a second episode, and 80-90% of those who have second episode will go on to have third one.[8] A 15 year follow up study showed that a fifth of depressives recovered and remained continuously well, three fifths recovered but had further episodes and a further fifth either committed suicide or were always incapacitated.[7]

Depression is a chronic recurrent disorder; with single depressive episodes (ICD-10 F32) being surprisingly rare and most people with depressive illness meeting the criteria for recurrent depressive disorder (ICD-10 F33).[9] However, people seldom seek help for their first attack of depression and the first treated episode is often the third or fourth actual episode. The delay occurs due to a combination of under recognition, with shopping for non medical cures, or a misdiagnosis leading to over investigation as well as searching for some elusive physical diagnosis. What is intriguing is that, often in the next recurrence, the patients do not seek psychiatric assistance, but go to other specialists depending on the presenting symptom, or go through another round of investigations to identify the problem. They neither link the present distress to their earlier episode, nor retain old prescriptions, nor refer to the earlier episode because of the stigma of the illness.

## BEHIND THE MASK

Patients who visited primary care physicians with the 10 most common symptoms were investigated and studied over 3 years.[10] The investigators found that a high proportion of the five most common symptoms: chest pain, fatigue, dizziness, headache and oedema, could not be identified as caused by organic illness. The percentages of the five next common symptoms i.e. backache, dyspnoea, insomnia, abdominal pain and numbness, that could not be traced to a known organic cause was higher.[10] The likelihood of these symptoms representing undiagnosed psychiatric illness, mainly depression, was very high, and it was found that large funds were spent on workups in pursuit of the elusive physical diagnosis.[10] Overutilisers of medical care services have a high lifetime incidence (68%) of depression[10] and this is important for public health administrators.

Depression as well as diabetes have chronic courses marked by periods without symptoms and by occasional emergencies. The UK prospective diabetes study showed the effectiveness of intensive follow up in preventing long term complications in diabetic patients.[11] In diabetic patients there would be an instant discussion on the chronic nature of the disease and the steps to manage it. However, in depression clinicians rarely broach the issue of the chronicity of the illness, and few patients want to hear about it.[4] Strong consideration should be given to maintaining long-term antidepressant therapy in several groups of patients especially those with a history of multiple episodes, recurrence within one year of stopping treatment, double depression (chronic dysthymia with additional more severe and pervasive episodes of major depression), onset of major depression after age 60 years, comorbid anxiety or substance abuse, or chronic medical condition worsened by depression.[12]

Depression is seldom well managed if one simply waits for the patient to initiate the next consultation. A model of practice in which patients seek help only when they deem it necessary, is inappropriate for an episodic but lifelong illness that affects hope and volition, reduces compliance and predisposes to suicide.[4] There needs to be an honest discussion with the patient as to long term prognosis. This would lead to better compliance, and recognition of the importance of regular treatment and follow up.

Teaching patients and their families to recognize early signs of the recurrence of depression and to train them in cognitive and behavioral techniques to deal with the prodromal phase may be another fruitful method.[13,14] In fact, some patients and families discover idiosyncratic early warning signs which occur like a ‘relapse signature’ and could help predict relapse. However, when patients do not take their drugs, do not implement their pleasant event activities, do not use problem solving routinely nor attend for appointments, they may benefit from a relapse prevention program.[4]

## STAYING OUT OF THE TRAP

A relapse prevention program can be set up by establishing a register of cases and proactively organizing appointments. Educating patient and family about depression and its recurrence, early warning signs, substance abuse, and use of medication as well as psychological approaches to aid recovery are of great importance.[4] Psychological approaches include getting oneself organized, seeking social support, distracting oneself from negative thoughts by doing things and recognizing and evaluating negative thoughts as well as avoid both staying in bed and wishing the problem to go away and taking extra medication without prescription and doing nothing.[13,14]

Intensive approaches in the program could include a rehearsal, an action plan for relapse prevention, and instruction on stress management, life style regularity i.e. maintenance of a healthy routine and of regular sleep, use of problem solving, communication training and cognitive therapy.[13,14] The benefits of relapse prevention programmes has been in delaying hospitalizations, shortened episode duration, better function; longer time to relapse, and fewer relapses, better compliance.[13,14]

Evidence based guidelines on depression have been formulated for our country by the Indian Psychiatric Society.[15] However, while these guidelines and those formulated elsewhere briefly dwell upon antidepressant maintenance therapy, they do not recommend a comprehensive relapse prevention programme, despite acknowledging the recurrent nature of depression.

Psychiatrists should learn from cardiologists, who encourage their patients to reduce their risk factors (including type A behavior) after a myocardial infarction. An aggressive attempt at relapse prevention, which includes cognitive and behavioural strategies, could help in reducing the burden of depression in patients and their families.
